# A Bio-Inspired Multi-Scale Adaptive Particle Filter for Scalar Gravity Matching Navigation in GNSS-Denied Underwater Environments

**DOI:** 10.3390/biomimetics11090651

**Published:** 2026-09-09

**Authors:** Xu Xia, Ningfang Song, Tianze Wang, Jian Guo, Jingchao Ban, Zhenpeng Wang

**Affiliations:** 1 School of Instrumentation and Optoelectronic Engineering, Beihang University, Beijing 100191, China; 2 Hunan Aerospace Electromechanical Equipment and Special Materials Research Institute, Changsha 410205, China; 3 College of Control and Information Engineering, Northeast Forestry University, Harbin 150040, China; 4 Sichuan Aerospace System Engineering Research Institute, Chengdu 610100, China

**Keywords:** gravity matching navigation, underwater autonomous vehicles, GNSS denial environment

## Abstract

In Global Navigation Satellite System (GNSS)-denied deep-sea environments, traditional scalar gravity matching navigation methods frequently suffer severe performance degradation in weak-feature, highly repetitive gravity anomaly regions. Inspired by the hippocampal spatial memory mechanism and natural graded foraging behavior of benthic marine organisms, this paper proposes a full-chain bionic framework named the Physics-Consistent Multi-Scale Adaptive Particle Filter for Gravity Matching Navigation (PC-MAPF-GM). This method endows the particle filter with four layers of biologically mimicked autonomous regulation capabilities: quantitative gravity field local suitability assessment, dynamically adjusted time-varying search scope, three-level multi-scale stepwise matching, and along-track trajectory motion physics consistency constraint. The verification of long-term shipborne lake experiments confirms that the proposed method reduces the final gravity matching positioning root mean square error (RMSE) to only 528.2 m, which is more than 41% lower than the classical terrain contour matching (TERCOM) benchmark and 31% lower than iterative closest contour point (ICCP). This biomimetic full-design-chain solution provides a robust new practical navigation paradigm for long-endurance fully autonomous underwater vehicles operating without any external auxiliary positioning information.

## 1. Introduction

Autonomous navigation in Global Navigation Satellite System (GNSS)-denied environments remains a fundamental challenge for long-duration underwater vehicles, polar platforms, and other autonomous systems operating in highly constrained environments [1,2,3]. Inertial navigation systems (INS) can provide continuous and self-contained positioning information; however, their errors inevitably accumulate over time due to sensor biases, scale-factor errors, and stochastic drift [4,5]. For underwater vehicles, surfacing to obtain GNSS fixes may be infeasible or undesirable because of mission continuity, concealment, and operational safety requirements. Therefore, developing passive, stable, and infrastructure-free navigation methods is of significant importance for autonomous underwater navigation [6].

Nature provides valuable inspiration for solving this problem. Many biological organisms achieve robust long-distance navigation by combining self-motion integration with environmental cues. Instead of relying on a single external positioning source, biological navigation systems often exploit naturally existing signals, such as magnetic fields [7], visual landmarks [8], odor gradients [9], optic flow [10], and other spatially distributed environmental features, to correct accumulated path-integration errors. This principle has motivated increasing interest in bio-inspired navigation systems for robots and autonomous platforms, where biologically inspired perception, information processing, and control strategies are transformed into engineering algorithms [11].

Inspired by such biological navigation principles, geophysical-field-aided navigation has emerged as a promising solution for autonomous vehicles operating without GNSS [12]. The Earth’s gravity anomaly field is spatially correlated, temporally stable, globally available, and difficult to jam or spoof. These properties make it a useful natural reference field for passive navigation [13]. In gravity matching navigation, the measured scalar gravity sequence along a vehicle trajectory is compared with a pre-stored gravity anomaly reference map, and the vehicle position is corrected according to the best-matched map location. From a biomimetic perspective, this process can be interpreted as an artificial analogue of environmental-field-based navigation: the vehicle integrates its motion through INS mechanization and periodically corrects its accumulated error by recognizing spatial patterns in a stable natural field.

Classical gravity matching methods include terrain contour matching (TERCOM), iterative closest contour point (ICCP), and Sandia inertial terrain-aided navigation (SITAN). TERCOM-type methods generally rely on correlation or MSD matching between measured and reference sequences [14]. ICCP-type methods use contour or equivalent-value features to iteratively reduce position errors [15], while SITAN-type methods formulate the problem within a filtering framework [16]. These methods have laid the foundation for gravity-aided inertial navigation and have been widely used as benchmark approaches in related studies. Nevertheless, several limitations remain unresolved when only scalar gravity measurements are available.

First, scalar gravity matching is highly sensitive to the local characteristics of the gravity anomaly field [17]. In regions with strong spatial variations, gravity measurements provide sufficient distinguishable information for position correction. However, in weak-feature or smooth regions, different candidate locations may exhibit similar gravity values, leading to ambiguous or false matches [18,19]. Traditional matching methods mainly evaluate residuals or correlations but usually do not explicitly quantify whether the local gravity field is suitable for reliable matching. As a result, a small residual may be incorrectly interpreted as a high-confidence match, even when the local field lacks sufficient spatial uniqueness [20].

Second, the search window in many existing methods is often empirically determined or fixed in advance. In practical navigation, however, INS uncertainty changes with time and mission conditions. A small search window may exclude the true position when INS drift becomes large, whereas an excessively large window increases computational burden and the probability of false matching [21]. Moreover, the appropriate search region should depend not only on INS uncertainty but also on the discriminability of the local gravity field [22]. This suggests that the matching search space should be jointly adapted according to navigation uncertainty and environmental-field suitability [23].

Third, scalar gravity measurements contain limited instantaneous information. If the matching likelihood is constructed only from single-point gravity residuals, the algorithm may be vulnerable to sensor noise, map errors, and local value similarity [24,25]. Biological navigation systems, in contrast, rarely rely on a single isolated cue; instead, they accumulate sequential evidence and exploit the consistency of environmental changes during movement [26]. Motivated by this principle, scalar gravity matching should make better use of the along-track gravity variation pattern rather than only the absolute gravity value at a single epoch.

Particle filtering provides a flexible Bayesian framework for nonlinear and non-Gaussian navigation problems. Compared with linearized filters, particle filters can represent multimodal posterior distributions and are therefore suitable for gravity matching in ambiguous environments [27]. However, a conventional particle filter that updates particle weights only according to scalar gravity residuals still suffers from particle degeneracy and false convergence in weak-feature regions [28]. To improve robustness, additional mechanisms are required to assess environmental suitability, regulate the search space, and impose physical consistency constraints on the matching process.

To address these issues, this paper proposes a Physics-Consistent Multi-Scale Adaptive Particle Filter for Scalar Gravity Matching Navigation, referred to as PC-MAPF-GM. The proposed method is designed for the practical condition in which only scalar gravity measurements are available. Its central idea is to imitate several useful principles observed in biological navigation: multi-scale environmental perception, suitability-aware cue selection, path-integration error correction, and sequential evidence accumulation. Specifically, a local gravity field suitability index is first constructed by integrating map-derived gradient magnitude, local roughness, information entropy, and matching uniqueness. This index is used to evaluate the reliability of gravity matching in different regions and is embedded into the particle weight update to suppress unreliable matches in weak-feature areas. Then, an adaptive search-window strategy is developed by jointly considering the INS error covariance and local gravity suitability, allowing the particle distribution to expand or contract according to both navigation uncertainty and environmental discriminability. Furthermore, an along-track gravity variation consistency constraint is introduced to exploit the physical continuity of scalar gravity observations, thereby reducing false matches caused by similar absolute gravity values. Finally, a multi-scale matching framework is incorporated to improve convergence robustness and avoid premature trapping in local optima.

Before detailing the proposed algorithm framework, we explicitly clarify the one-to-one logical mapping from bionic navigation prototype to each algorithm module in this work, as summarized in Table 1.

To further highlight the novelty of this full-chain bionic design, we provide a systematic comparative review of representative recent bio-inspired gravity matching navigation works in Table 2.

The main contributions of this paper are summarized as follows:

(1) A suitability-aware scalar gravity matching framework is developed for GNSS-denied underwater navigation. A quantitative gravity-field suitability evaluation model is constructed by integrating the local gradient, roughness, entropy, and spatial uniqueness of the gravity anomaly map. This model explicitly characterizes the matching capability of different regions and provides an environmental information basis for adaptive navigation correction.
(2) A physics-constrained multi-scale adaptive particle filter is proposed. The particle search region is dynamically adjusted according to both INS position uncertainty and local gravity-field suitability, thereby avoiding the limitations of fixed search windows. In addition, multi-scale gravity maps are introduced to construct a sequence-based residual, which combines absolute gravity anomaly consistency with along-track gravity variation constraints to improve robustness against measurement noise, map errors, and local field ambiguity.
(3) An adaptive weight modulation and resampling strategy is designed to enhance matching reliability. The particle weights are updated by jointly considering the multi-scale matching residual and the local suitability index, while systematic resampling with suitability-dependent roughening is employed to alleviate particle degeneracy. Simulation results demonstrate that the proposed method can effectively suppress INS error accumulation and achieve more accurate and stable positioning than conventional INS-only navigation in complex gravity anomaly environments.

The remainder of this paper is organized as follows: Section 2 formulates the scalar gravity matching navigation problem and analyzes the limitations of conventional residual-based matching methods. Section 3 presents the proposed Physics-Consistent Multi-Scale Adaptive Particle Filter for Gravity Matching Navigation (PC-MAPF-GM), including gravity-field suitability evaluation, adaptive search-region construction, multi-scale likelihood modeling, and suitability-aware particle weight updating. Section 4 describes the simulation and shipboard experimental settings and discusses the corresponding navigation performance. Finally, Section 5 concludes the paper.

## 2. Problem Statement

### 2.1. Bio-Inspired View of Environmental-Field-Based Navigation

Many biological navigation systems do not rely on a single absolute positioning source. Instead, animals often combine self-motion integration with environmental cues to achieve robust navigation over long distances. In such systems, path integration provides a continuous estimate of motion, while stable environmental features are used to correct accumulated drift. This principle has inspired robotic navigation methods that integrate proprioceptive dead reckoning with external natural cues, such as visual landmarks, geomagnetic fields, odor gradients, or other spatially distributed environmental signatures.

Scalar gravity matching navigation follows a similar information-processing paradigm. The inertial navigation system provides a continuous but drifting estimate of the vehicle trajectory, while the gravity anomaly field provides a stable natural reference. By comparing the measured gravity sequence with a pre-stored gravity anomaly map, the accumulated INS error can be constrained. From a bio-inspired perspective, the gravity anomaly map can be regarded as an environmental field memory, and the matching process can be interpreted as environmental-cue-based correction of path-integration drift.

In the paper, we focus on the practical condition where only scalar gravity measurements are available. No gravity gradient tensor, bathymetric observation, geomagnetic measurement, or acoustic positioning information is assumed. Therefore, the main challenge is to extract sufficient positional information from a one-dimensional scalar gravity sequence and a two-dimensional gravity anomaly reference map.

### 2.2. Coordinate Definition and State Representation

The horizontal position of an underwater vehicle at time step k is denoted by

(1) $$p_{k}={\left[\begin{array}{ll} x_{k} & y_{k} \end{array}\right]}^{T},$$

where $x_{k}$ and $y_{k}$ represent the east and north coordinates in a local-level navigation frame. For regional navigation problems, longitude and latitude can also be converted into local Cartesian coordinates by map projection. Since the gravity matching process mainly corrects horizontal position errors, this study focuses on two-dimensional horizontal positioning.

The INS-predicted position is denoted by

(2) $${\hat{p}}_{\mathrm{INS},k}={\left[\begin{array}{ll} {\hat{x}}_{\mathrm{INS},k} & {\hat{y}}_{\mathrm{INS},k} \end{array}\right]}^{T}.$$

In practice, the INS prediction error gradually increases with time. The horizontal INS error covariance matrix is written as

(3) $$P_{\mathrm{INS},k}=\left[\begin{array}{cc} \sigma_{x,k}^{2} & \sigma_{xy,k}\\ \sigma_{xy,k} & \sigma_{y,k}^{2} \end{array}\right].$$

The gravity matching algorithm aims to estimate the corrected position

(4) $${\hat{p}}_{k}={\left[\begin{array}{ll} {\hat{x}}_{k} & {\hat{y}}_{k} \end{array}\right]}^{T}$$

By jointly using the INS prediction, scalar gravity measurements, and the gravity anomaly reference map.

### 2.3. INS-Based Motion Prediction Model

The vehicle motion between two adjacent epochs can be approximately represented by the displacement increment derived from INS mechanization:

(5) $$\Delta s_{k}={\left[\begin{array}{ll} \Delta x_{k} & \Delta y_{k} \end{array}\right]}^{T}.$$

Then, the nominal position propagation model is

(6) $$p_{k}=p_{k-1}+\Delta s_{k}+w_{k},$$

where $w_{k}$ is the process noise accounting for INS drift, velocity error, heading error, and unmodeled dynamic disturbances. In a particle filtering framework, the i-th particle is propagated as

(7) $$p_{k}^{\left(i\right)}=p_{k-1}^{\left(i\right)}+\Delta s_{k}+w_{k}^{\left(i\right)},i=1,2,\ldots ,N,$$

where N is the number of particles.

The process noise is commonly modeled as a zero-mean Gaussian random vector:

(8) $$w_{k}^{\left(i\right)}\sim N\left(0,Q_{k}\right),$$

where $Q_{k}$ is determined according to the INS error characteristics and vehicle motion uncertainty. In the proposed framework, the INS error covariance is not only used for particle propagation but also for adaptive search-window determination, which will be described in Section 3.

### 2.4. Scalar Gravity Observation Model

Let the scalar gravity anomaly measured by the onboard gravimeter at time step k be denoted as

(9) $$z_{k}=\Delta g_{\mathrm{obs},k}.$$

The corresponding gravity anomaly value at position $p_{k}$ in the reference map is

(10) $$m\left(p_{k}\right)=\Delta g_{\mathrm{map}}\left(p_{k}\right),$$

where m(⋅) represents the gravity anomaly reference map. Since the particle position may not coincide with a grid node of the reference map, bilinear interpolation or bicubic interpolation is usually required to obtain the map value at an arbitrary candidate position.

The scalar gravity observation model can be written as

(11) $$z_{k}=m\left(p_{k}\right)+v_{k},$$

where $v_{k}$ is the scalar gravity measurement error, including gravimeter noise, map error, temporal variation residuals, and preprocessing errors. It is commonly assumed that

(12) $$v_{k}\sim N\left(0,\sigma_{g}^{2}\right),$$

where $\sigma_{g}^{2}$ is the equivalent variance of scalar gravity observation uncertainty.

For the i-th particle, the single-point gravity residual is

(13) $$r_{k}^{\left(i\right)}=z_{k}-m\left(p_{k}^{\left(i\right)}\right)$$

The corresponding single-point likelihood can be expressed as

(14) $$p\left(z_{k}|p_{k}^{\left(i\right)}\right)\propto \exp\left[-\frac{{\left(r_{k}^{\left(i\right)}\right)}^{2}}{2\sigma_{g}^{2}}\right].$$

However, single-point scalar gravity matching is often insufficient because different positions may have similar gravity anomaly values, especially in weak-feature regions. Therefore, sequence-based matching is generally more reliable.

### 2.5. Sequence-Based Scalar Gravity Matching

A sliding gravity sequence of length L is considered:

(15) $$z_{k,L}={\left[\begin{array}{llll} z_{k-L+1} & z_{k-L+2} & \cdots & z_{k} \end{array}\right]}^{T}$$

For the i-th particle trajectory, the corresponding map-derived gravity sequence is

(16) $$m_{k,L}^{\left(i\right)}={\left[\begin{array}{llll} m\left(p_{k-L+1}^{\left(i\right)}\right) & m\left(p_{k-L+2}^{\left(i\right)}\right) & \cdots & m\left(p_{k}^{\left(i\right)}\right) \end{array}\right]}^{T}.$$

The sequence residual vector is defined as

(17) $$r_{k,L}^{\left(i\right)}=z_{k,L}-m_{k,L}^{\left(i\right)}.$$

A commonly used sequence-matching cost is the mean square residual:

(18) $$D_{g,k}^{\left(i\right)}=\frac{1}{L}\sum_{j=k-L+1}^{k}{\left[z_{j}-m\left(p_{j}^{\left(i\right)}\right)\right]}^{2}$$

The corresponding sequence-based likelihood is

(19) $$p\left(z_{k,L}|p_{k}^{\left(i\right)}\right)\propto \exp\left[-\frac{D_{g,k}^{\left(i\right)}}{2\sigma_{g}^{2}}\right].$$

### 2.6. Conventional Particle-Filter-Based Gravity Matching

The standard particle filtering procedure consists of three main steps: prediction, weight update, and resampling.

First, particles are propagated using the INS-derived motion increment:

(20) $$p_{k}^{\left(i\right)}\sim p\left(p_{k}|p_{k-1}^{\left(i\right)}\right)$$

Second, the particle weights are updated according to the scalar gravity matching likelihood:

(21) $${\tilde{w}}_{k}^{\left(i\right)}=w_{k-1}^{\left(i\right)}p\left(z_{k,L}|p_{k}^{\left(i\right)}\right),$$

where ${\tilde{w}}_{k}^{\left(i\right)}$ is the unnormalized weight. Using the sequence residual defined in Equation (18), the conventional weight update becomes

(22) $${\tilde{w}}_{k}^{\left(i\right)}=w_{k-1}^{\left(i\right)}\exp\left[-\frac{D_{g,k}^{\left(i\right)}}{2\sigma_{g}^{2}}\right].$$

Then, the weights are normalized as

(23) $$w_{k}^{\left(i\right)}=\frac{{\tilde{w}}_{k}^{\left(i\right)}}{\sum_{j=1}^{N}{\tilde{w}}_{k}^{\left(j\right)}}.$$

Finally, the estimated position is obtained by weighted averaging:

(24) $${\hat{p}}_{k}=\sum_{i=1}^{N}w_{k}^{\left(i\right)}p_{k}^{\left(i\right)}$$

To avoid particle degeneracy, the effective number of particles is calculated as

(25) $$N_{\mathrm{eff}}=\frac{1}{\sum_{i=1}^{N}{\left(w_{k}^{\left(i\right)}\right)}^{2}}$$

When $N_{\mathrm{eff}\;}$ is smaller than a predefined threshold $N_{\mathrm{th}\;}$, resampling is performed.

Although this framework is flexible and theoretically suitable for nonlinear gravity matching problems, the conventional particle-filter-based method still mainly depends on residual minimization. Therefore, particles located in weak-feature regions may obtain high weights if their scalar gravity values happen to be similar to the measured values, even when the match is physically unreliable.

### 2.7. Limitations of Existing Scalar Gravity Matching Methods

When only scalar gravity information is available, gravity matching navigation faces several fundamental difficulties.

#### 2.7.1. Ambiguity in Weak-Feature Gravity Regions

The amount of useful information contained in scalar gravity measurements depends strongly on the spatial variability in the gravity anomaly field. In a region where the gravity field changes significantly, small position deviations can lead to observable gravity differences. In contrast, in a smooth region, many candidate positions may produce nearly identical gravity values. This reduces the discriminability of scalar gravity matching.

Let two candidate positions be $p_{a}$ and $p_{b}$. If

(26) $$m\left(p_{a}\right)\approx m\left(p_{b}\right)$$

Or more generally,

(27) $$m_{k,L}\left(p_{a}\right)\approx m_{k,L}\left(p_{b}\right),$$

Then, gravity matching may fail to distinguish between these two candidate locations. This problem is particularly serious in flat gravity regions or regions with repeated anomaly patterns.

Traditional residual-based likelihood functions do not explicitly evaluate the matchability of the local gravity field. Therefore, a small residual does not necessarily imply a reliable position estimate.

#### 2.7.2. Fixed or Empirically Defined Search Window

Many matching methods require a predefined search region around the INS-predicted position. However, the INS error grows with time and varies with sensor quality, vehicle dynamics, and mission duration. A fixed search window is therefore not optimal.

If the search window is too small, the true position may be excluded:

(28) $$p_{k}^{\mathrm{true}\;}\notin \Omega_{k},$$

where $\Omega_{k}$ denotes the search region. In this case, no matching algorithm can recover the correct position.

If the search window is too large, the number of false candidate positions increases, and the computational cost also becomes higher. Moreover, in weak-feature areas, a large search window can significantly increase the probability of false matching. Therefore, the search region should be adaptively determined according to both INS uncertainty and local gravity-field discriminability.

#### 2.7.3. Insufficient Use of Sequential Physical Consistency

Scalar gravity observations are not independent isolated values along the trajectory. Since the gravity anomaly field is spatially continuous, consecutive gravity measurements should exhibit physically consistent variations with vehicle motion. However, many existing methods mainly use absolute gravity residuals or correlation coefficients, without sufficiently exploiting along-track variation consistency.

For example, two candidate trajectories may have similar absolute gravity values but different gravity variation trends. If only the residual magnitude is considered, these two candidates may be difficult to distinguish. Therefore, incorporating the consistency between measured and map-derived gravity variation can provide additional constraints for scalar gravity matching.

In this work, the abbreviation PC (Physics-Consistent) explicitly refers to the kinematic motion physical constraint for underwater vehicles: it strictly limits the maximum one-step position jump of any sampled particle to ≤1.2 times the maximum cruising speed of the tested underwater vehicle, which eliminates all impossible pseudo-matching points that violate basic motion physics, and this design is inherently mimicking the natural continuous movement property where benthic organisms never teleport abruptly on the seabed.

#### 2.7.4. Local Convergence and Particle Degeneracy

In conventional particle filtering, particles with relatively small residuals receive large weights. In complex or ambiguous gravity fields, this may cause the particle set to converge prematurely to a false location. Once most particles concentrate in an incorrect region, subsequent resampling may eliminate particles near the true position, leading to irreversible divergence.

This issue is analogous to unreliable cue dominance in biological navigation: when an environmental cue is ambiguous, over-trusting it can degrade navigation performance. A more robust strategy should evaluate cue reliability before using it for strong correction. In scalar gravity matching, this means that the algorithm should not only measure the residual between observation and map but also assess whether the local gravity field provides sufficient information for reliable matching.

Based on the above analysis, the problem addressed in this paper can be formulated as follows.

Given the following:

1. The INS-predicted position sequence

(29) $${\left\{{\hat{p}}_{\mathrm{INS},k}\right\}}_{k=1}^{K},$$

2. The INS error covariance matrix

(30) $$P_{\mathrm{INS}\;,k},$$

3. The scalar gravity measurement sequence

(31) $${\left\{z_{k}\right\}}_{k=1}^{K},$$

4. The scalar gravity anomaly reference map

(32) m(p),

the objective is to estimate the corrected vehicle position sequence

(33) $${\left\{{\hat{p}}_{k}\right\}}_{k=1}^{K}$$

Such that the positioning error

(34) $$e_{k}=\left\|{\hat{p}}_{k}-p_{k}^{\mathrm{true}\;}\right\|$$

is minimized over the navigation process.

More specifically, this study aims to design a scalar-gravity-only matching method that satisfies the following requirements:

1. Reliability in weak-feature regions: the method should reduce false matching when the local gravity field has low spatial discriminability.
2. Adaptability to INS uncertainty: the search range should be adjusted according to the time-varying INS error covariance.
3. Use of sequential gravity information: the method should exploit not only absolute scalar gravity values but also along-track gravity variation consistency.
4. Robust Bayesian estimation: the method should maintain multiple candidate hypotheses before sufficient evidence is accumulated, thereby reducing premature convergence.

To meet these requirements, the next section proposes a Physics-Consistent Multi-Scale Adaptive Particle Filter for Scalar Gravity Matching Navigation, abbreviated as PC-MAPF-GM.

## 3. Proposed Method

### 3.1. Framework of the Proposed PC-MAPF-GM Method

The PC-MAPF-GM framework proposed in the study is entirely generated by biomimetic mapping of the natural navigation behavior of multiple types of benthic marine organisms: the gravity field local fitness modeling module directly simulates the pre assessment behavior of benthic organisms on food density and habitat suitability in the seabed area before foraging, corresponding to the encoding process of “environmental navigability” in the spatial memory of the hippocampus; adaptive time-varying search interval module, replicating the graded foraging strategy of organisms enlarging the exploration range in unfamiliar areas and reducing the search step size in familiar high-resolution feature areas; multiscale step-by-step matching module, corresponding to the hierarchical spatial recognition behavior of organisms gradually focusing from large-scale coarse positioning to small-scale precise foraging points; the physical consistency constraint module along the trajectory directly simulates the natural physical properties of biological continuous cruising without spatial teleportation. This full chain biomimetic design ensures that every core component of the algorithm has a clear biological mechanism traceability, rather than simply engineering heuristic improvements.

To improve the robustness of scalar gravity matching navigation in GNSS-denied underwater environments, this paper proposes a Physics-Consistent Multi-Scale Adaptive Particle Filter for Gravity Matching Navigation, abbreviated as PC-MAPF-GM. The method is designed for the practical condition in which only scalar gravity observations are available. It does not require gravity gradient tensor measurements, bathymetric information, geomagnetic measurements, or external acoustic positioning.

The proposed method follows a bio-inspired environmental-field-based navigation philosophy. In biological navigation, self-motion estimation is often corrected by stable environmental cues, but such cues are not used blindly. Their reliability, spatial distinctiveness, and temporal consistency are implicitly evaluated during movement. Inspired by this principle, the proposed method treats INS mechanization as an engineering counterpart of path integration, while the gravity anomaly map is regarded as a stable environmental field memory. Scalar gravity measurements are then used as environmental cues to correct accumulated INS drift.

In conventional particle-filter-based gravity matching, particle weights are usually determined mainly by the residual between measured scalar gravity values and map-derived scalar gravity values. However, this residual-only mechanism may be unreliable in weak-feature regions, where many candidate positions can produce similar scalar gravity values. Therefore, PC-MAPF-GM introduces three coupled mechanisms into the particle filtering framework. First, a gravity field suitability index is constructed from the reference gravity anomaly map to quantify the local matchability of scalar gravity information. Second, an adaptive search region is determined by jointly considering INS uncertainty and gravity field suitability. Third, a physics-consistent multi-scale gravity matching likelihood is established by combining sequence similarity, along-track gravity variation consistency, and multi-scale map information.

The overall workflow is as follows: At each time step, the INS-derived displacement is used to propagate the particles. Around each candidate position, scalar gravity values are interpolated from the reference map, and the measured gravity sequence is compared with the map-derived sequence. Meanwhile, the local suitability of the gravity field is evaluated. The final particle weight is determined not only by gravity sequence similarity, but also by the reliability of the local gravity field and the physical consistency of along-track gravity variation. After normalization and resampling, the corrected position is obtained from the weighted particle set.

### 3.2. Gravity Field Suitability Modeling and Adaptive Search Region

The reliability of scalar gravity matching depends strongly on the spatial characteristics of the gravity anomaly field. In areas with strong spatial variation and distinctive local patterns, scalar gravity measurements can provide effective positional information. In contrast, in smooth or repetitive regions, different candidate positions may produce similar gravity values or similar gravity sequences, leading to false matching. Therefore, before scalar gravity information is used for strong position correction, the local gravity field should be evaluated in terms of its suitability for matching.

Let m(p) denote the scalar gravity anomaly value at position $p={\left[x,y\right]}^{T}$, and let Ω(p) be a local window centered at p. In this study, the gravity field suitability index is constructed by integrating four map-derived components: gradient magnitude, local roughness, information entropy, and matching uniqueness.

The first component is the spatial gradient magnitude of the gravity anomaly map:

(35) $$G_{1}(p)=\sqrt{{\left(\frac{\partial m}{\partial x}\right)}^{2}+{\left(\frac{\partial m}{\partial y}\right)}^{2}}.$$

The partial derivatives are calculated from the reference map by finite differences:

(36) $$\frac{\partial m}{\partial x}\approx \frac{m(x+\Delta x,y)-m(x-\Delta x,y)}{2\Delta x}$$

(37) $$\frac{\partial m}{\partial y}\approx \frac{m(x,y+\Delta y)-m(x,y-\Delta y)}{2\Delta y}$$

A larger value of $G_{1}(p)$ indicates that a small position change can produce a more observable gravity difference, which is beneficial for scalar gravity matching.

The second component is the local gravity roughness, defined as the standard deviation of gravity anomaly values within the local window:

(38) $$G_{2}(p)=\sqrt{\frac{1}{M}\sum_{q\in \Omega (p)}{\left[m(q)-{\bar{m}}_{\Omega }\right]}^{2}},$$

where M is the number of grid points in the local window and

(39) $${\bar{m}}_{\Omega }=\frac{1}{M}\sum_{q\in \Omega (p)}m(q).$$

A larger roughness value usually implies stronger local fluctuation and better spatial discriminability.

The third component is the local information entropy. The gravity anomaly values in Ω(p) are divided into B bins, and the probability of the b-th bin is denoted by $P_{b}$. The entropy is calculated as

(40) $$G_{3}(p)=-\sum_{b=1}^{B}P_{b}\log\left(P_{b}+\varepsilon \right),$$

where ε is a small positive constant used to avoid numerical instability. A high entropy value means that the local gravity distribution contains richer information, whereas a low entropy value often corresponds to a smooth or homogeneous region.

The fourth component is the local matching uniqueness. Even if a local gravity pattern has significant variation, it may still be similar to nearby patterns, causing ambiguous matching. Let $\gamma_{0}(p)$ denote the local gravity pattern centered at p, and let $\gamma_{j}(p)$ denote a neighboring pattern shifted by a nonzero displacement. If $\rho_{j}(p)$ is the normalized correlation coefficient between these two patterns, the uniqueness component is defined as

(41) $$G_{4}(p)=1-\max_{j\neq 0}\left|\rho_{j}(p)\right|.$$

A large $G_{4}(p)$ indicates that the local gravity pattern is less similar to its surroundings and is therefore more suitable for reliable matching.

Since the four components have different dimensions and numerical ranges, each component is normalized into [0,1]:

(42) $${\tilde{G}}_{l}(p)=\frac{G_{l}(p)-G_{l}^{\min}}{G_{l}^{\max}-G_{l}^{\min}+\varepsilon },l=1,2,3,4.$$

The final suitability index is obtained by weighted fusion:

(43) $$A(p)=\alpha_{1}{\tilde{G}}_{1}(p)+\alpha_{2}{\tilde{G}}_{2}(p)+\alpha_{3}{\tilde{G}}_{3}(p)+\alpha_{4}{\tilde{G}}_{4}(p),$$

where

(44) $$\sum_{l=1}^{4}\alpha_{l}=1,\alpha_{l}\geq 0$$

The value of A(p) lies in [0,1]. A larger value means that the local gravity field is more informative and more suitable for scalar gravity matching.

The suitability index is further used to define the adaptive search region. At time step k, the INS provides the predicted position ${\hat{p}}_{INS,k}$ and the horizontal error covariance matrix $P_{INS,k}$. The INS uncertainty radius is defined using the largest eigenvalue of the covariance matrix:

(45) $$R_{\mathrm{INS},k}=\kappa \sqrt{\lambda_{\max}\left(P_{\mathrm{INS},k}\right)},$$

where $\lambda_{\max}(\cdot )$ denotes the maximum eigenvalue and κ is a confidence coefficient.

To incorporate gravity field reliability, the average suitability around the INS-predicted position is calculated as

(46) $${\bar{A}}_{k}=\frac{1}{\left|\Omega_{\mathrm{INS},k}\right|}\int_{\Omega_{\mathrm{NS},k}}A(p)dp$$

where $\Omega_{\mathrm{INS},\;k}$ is a local neighborhood centered at ${\hat{p}}_{\mathrm{INS},\;k}$. In discrete implementation, the integral is replaced by the average over grid points or sampled candidate positions.

The adaptive search radius is then defined as

(47) $$R_{k}=R_{\mathrm{INS},k}\left[1+\mu \left(1-{\bar{A}}_{k}\right)\right]$$

where μ≥0 is a suitability-based expansion coefficient. When the local suitability is high, the search radius remains close to the INS uncertainty radius. When the local suitability is low, the search region is enlarged to preserve multiple possible hypotheses and reduce premature convergence.

The adaptive search region is therefore given by

(48) $$\Omega_{k}=\left\{p:\left\|p-{\hat{p}}_{\mathrm{INS}\;,k}\right\|\leq R_{k}\right\}$$

This design links navigation uncertainty with environmental-field reliability. It prevents the true position from being excluded when INS uncertainty grows, while also avoiding overconfident matching in weak-feature gravity regions.

### 3.3. Physics-Consistent Multi-Scale Gravity Matching Likelihood

After particle propagation, each particle represents a candidate vehicle position. For the i-th particle at time step k, the map-derived scalar gravity value is obtained by interpolation:

(49) $${\hat{z}}_{k}^{\left(i\right)}=m\left(p_{k}^{\left(i\right)}\right).$$

To reduce the ambiguity of single-point scalar matching, a sliding gravity sequence of length L is used. The measured gravity sequence is written as

(50) $$z_{k,L}={\left[z_{k-L+1},z_{k-L+2},\ldots ,z_{k}\right]}^{T}$$

And the corresponding map-derived sequence for the i-th particle trajectory is

(51) $${\hat{z}}_{k,L}^{\left(i\right)}={\left[m\left(p_{k-L+1}^{\left(i\right)}\right),m\left(p_{k-L+2}^{\left(i\right)}\right),\ldots ,m\left(p_{k}^{\left(i\right)}\right)\right]}^{T}$$

The sequence matching residual is defined as

(52) $$D_{g,k}^{\left(i\right)}=\frac{1}{L}\sum_{j=k-L+1}^{k}{\left[z_{j}-m\left(p_{j}^{\left(i\right)}\right)\right]}^{2}$$

If the scalar gravity measurement noise is modeled as zero-mean Gaussian noise with variance $\sigma_{g}^{2}$, the basic sequence likelihood can be expressed as

(53) $$L_{g,k}^{\left(i\right)}=\exp\left(-\frac{D_{g,k}^{\left(i\right)}}{2\sigma_{g}^{2}}\right)$$

However, scalar gravity observations along a trajectory are not independent isolated values. Because the gravity anomaly field is spatially continuous, the variation trend of consecutive scalar gravity measurements should be physically consistent with the variation trend predicted from the reference map along the candidate trajectory. Therefore, an along-track gravity variation consistency term is introduced.

Let the traveled distance between time steps k−1 and k be

(54) $$\Delta s_{k}=\left\|{\hat{p}}_{\mathrm{INS},k}-{\hat{p}}_{\mathrm{INS},k-1}\right\|.$$

The observed along-track scalar gravity variation rate is defined as

(55) $${\dot{g}}_{\mathrm{obs},k}=\frac{z_{k}-z_{k-1}}{\Delta s_{k}+\varepsilon }$$

For the i-th particle trajectory, the map-derived along-track variation rate is

(56) $${\dot{g}}_{\mathrm{map},k}^{\left(i\right)}=\frac{m\left(p_{k}^{\left(i\right)}\right)-m\left(p_{k-1}^{\left(i\right)}\right)}{\Delta s_{k}+\varepsilon }$$

The physical consistency residual is then given by

(57) $$D_{p,k}^{\left(i\right)}={\left[{\dot{g}}_{\mathrm{obs},k}-{\dot{g}}_{\mathrm{map},k}^{\left(i\right)}\right]}^{2}$$

This term helps distinguish candidate trajectories that have similar absolute gravity values but different gravity variation trends. The combined physics-consistent residual is written as

(58) $$D_{k}^{\left(i\right)}=D_{g,k}^{\left(i\right)}+\lambda_{p}D_{p,k}^{\left(i\right)}$$

where $\lambda_{p}$ is a nonnegative coefficient controlling the contribution of the along-track consistency constraint.

To further improve robustness, the proposed method adopts a multi-scale matching strategy. Gravity anomaly maps contain spatial information at different scales. Coarse-scale maps are less sensitive to noise and local map errors, while fine-scale maps provide higher positioning accuracy when the gravity field is sufficiently informative. Let the multi-scale gravity maps be denoted as

(59) $$\left\{M^{\left(1\right)},M^{\left(2\right)},\ldots ,M^{\left(S\right)}\right\}$$

where $M^{\left(1\right)}$ is the coarsest map and $M^{\left(S\right)}$ is the finest map. The gravity anomaly value at scale s is denoted by $m^{\left(s\right)}(p)$.

At scale s, the sequence residual and physical consistency residual are respectively defined as

(60) $$D_{g,k}^{\left(i,s\right)}=\frac{1}{L}\sum_{j=k-L+1}^{k}{\left[z_{j}^{\left(s\right)}-m^{\left(s\right)}\left(p_{j}^{\left(i\right)}\right)\right]}^{2}$$

(61) $$D_{p,k}^{\left(i,s\right)}={\left[{\dot{g}}_{\mathrm{obs},k}^{\left(s\right)}-{\dot{g}}_{\mathrm{map},k}^{\left(i,s\right)}\right]}^{2}.$$

The multi-scale residual is then constructed as

(62) $$D_{\mathrm{ms},k}^{\left(i\right)}=\sum_{s=1}^{S}\beta_{s}\left[D_{g,k}^{\left(i,s\right)}+\lambda_{p}D_{p,k}^{\left(i,s\right)}\right]$$

where $\beta_{s}$ is the scale weight satisfying

(63) $$\sum_{s=1}^{S}\beta_{s}=1,\beta_{s}\geq 0$$

The scale weights can be determined according to the suitability of each scale. Let ${\bar{A}}_{k}^{\left(s\right)}$ be the average suitability at scale s. Then,

(64) $$\beta_{s}=\frac{{\bar{A}}_{k}^{\left(s\right)}}{\sum_{r=1}^{S}{\bar{A}}_{k}^{\left(r\right)}+\varepsilon }$$

Thus, the scale that contains more reliable gravity information contributes more to the final likelihood. The resulting physics-consistent multi-scale likelihood is

(65) $$L_{\mathrm{ms},k}^{\left(i\right)}=\exp\left(-\frac{D_{\mathrm{ms},k}^{\left(i\right)}}{2\sigma_{g}^{2}}\right)$$

This likelihood integrates three types of information: scalar gravity sequence similarity, along-track physical consistency, and multi-scale environmental-field structure. It therefore provides a more reliable evidence model than residual-only scalar gravity matching.

### 3.4. Suitability-Aware Particle Filtering Implementation

The proposed likelihood is embedded into a particle filtering framework to estimate the corrected vehicle position. At time step k, the posterior distribution of the vehicle position is approximated by a set of weighted particles:

(66) $$p\left(p_{k}|z_{1:k}\right)\approx \sum_{i=1}^{N}w_{k}^{\left(i\right)}\delta \left(p_{k}-p_{k}^{\left(i\right)}\right)$$

where N is the number of particles, $p_{k}^{\left(i\right)}$ is the position of the i-th particle, and $w_{k}^{\left(i\right)}$ is its normalized weight.

Particles are first propagated using the INS-derived displacement increment:

(67) $$p_{k}^{\left(i\right)}=p_{k-1}^{\left(i\right)}+\Delta s_{k}+w_{k}^{\left(i\right)},i=1,2,\ldots ,N,$$

where $\Delta s_{k}$ is the displacement increment obtained from INS mechanization, and $w_{k}^{\left(i\right)}$ is the process noise. The process noise is modeled as

(68) $$w_{k}^{\left(i\right)}\sim N\left(0,Q_{k}\right).$$

Particles outside the adaptive search region $\Omega_{k}$ are rejected or regenerated to ensure that the particle set remains consistent with INS uncertainty and the current search range.

The key step is the suitability-aware weight update. For the i-th particle, the local suitability value is

(69) $$A_{k}^{\left(i\right)}=A\left(p_{k}^{\left(i\right)}\right)$$

The unnormalized particle weight is updated as

(70) $${\tilde{w}}_{k}^{\left(i\right)}=w_{k-1}^{\left(i\right)}L_{\mathrm{ms},k}^{\left(i\right)}{\left[\epsilon_{A}+A_{k}^{\left(i\right)}\right]}^{\eta },$$

where $\epsilon_{A}$ is a small positive constant and η is the suitability regulation coefficient. Substituting Equation (66) into Equation (71), the weight update becomes

(71) $${\tilde{w}}_{k}^{\left(i\right)}=w_{k-1}^{\left(i\right)}\exp\left(-\frac{D_{\mathrm{ms},k}^{\left(i\right)}}{2\sigma_{g}^{2}}\right){\left[\epsilon_{A}+A\left(p_{k}^{\left(i\right)}\right)\right]}^{\eta }.$$

When η=0, the suitability index does not affect the particle weights, and the method reduces to a physics-consistent multi-scale particle filter. When η>0, particles located in informative gravity regions are strengthened, whereas particles in weak-feature regions are prevented from obtaining excessively large weights only because of accidental residual similarity. This design does not discard weak feature regions completely; instead, it reduces overconfidence in unreliable environmental cues.

The particle weights are normalized as

(72) $$w_{k}^{\left(i\right)}=\frac{{\tilde{w}}_{k}^{\left(i\right)}}{\sum_{j=1}^{N}{\tilde{w}}_{k}^{\left(j\right)}}$$

To monitor particle degeneracy, the effective number of particles is calculated as

(73) $$N_{\mathrm{eff},k}=\frac{1}{\sum_{i=1}^{N}{\left(w_{k}^{\left(i\right)}\right)}^{2}}.$$

When $N_{\mathrm{eff}\;,k}$ is smaller than a predefined threshold $N_{\mathrm{th}}$, resampling is performed. To preserve particle diversity after resampling, a small roughening noise can be added:

(74) $$p_{k}^{\left(i\right)}\leftarrow p_{k}^{\left(i\right)}+\xi_{k}^{\left(i\right)},$$

where

(75) $$\xi_{k}^{\left(i\right)}\sim N\left(0,Q_{r,k}\right).$$

The covariance $Q_{r,k}$ can be set according to map resolution, particle spread, and INS uncertainty. In weak-feature regions, a slightly larger roughening noise helps preserve alternative hypotheses; in strong-feature regions, a smaller roughening noise is preferred to improve convergence accuracy.

Finally, the corrected position is estimated as the weighted mean of the particle set:

(76) $${\hat{p}}_{k}=\sum_{i=1}^{N}w_{k}^{\left(i\right)}p_{k}^{\left(i\right)}.$$

The correction relative to the INS-predicted position is

(77) $$\delta {\hat{p}}_{k}={\hat{p}}_{k}-{\hat{p}}_{\mathrm{INS}\;,k}$$

This correction can be directly used as the gravity-aided navigation output or further integrated into an INS filtering system.

### 3.5. Algorithm Summary and Computational Analysis

**Figure 1:**
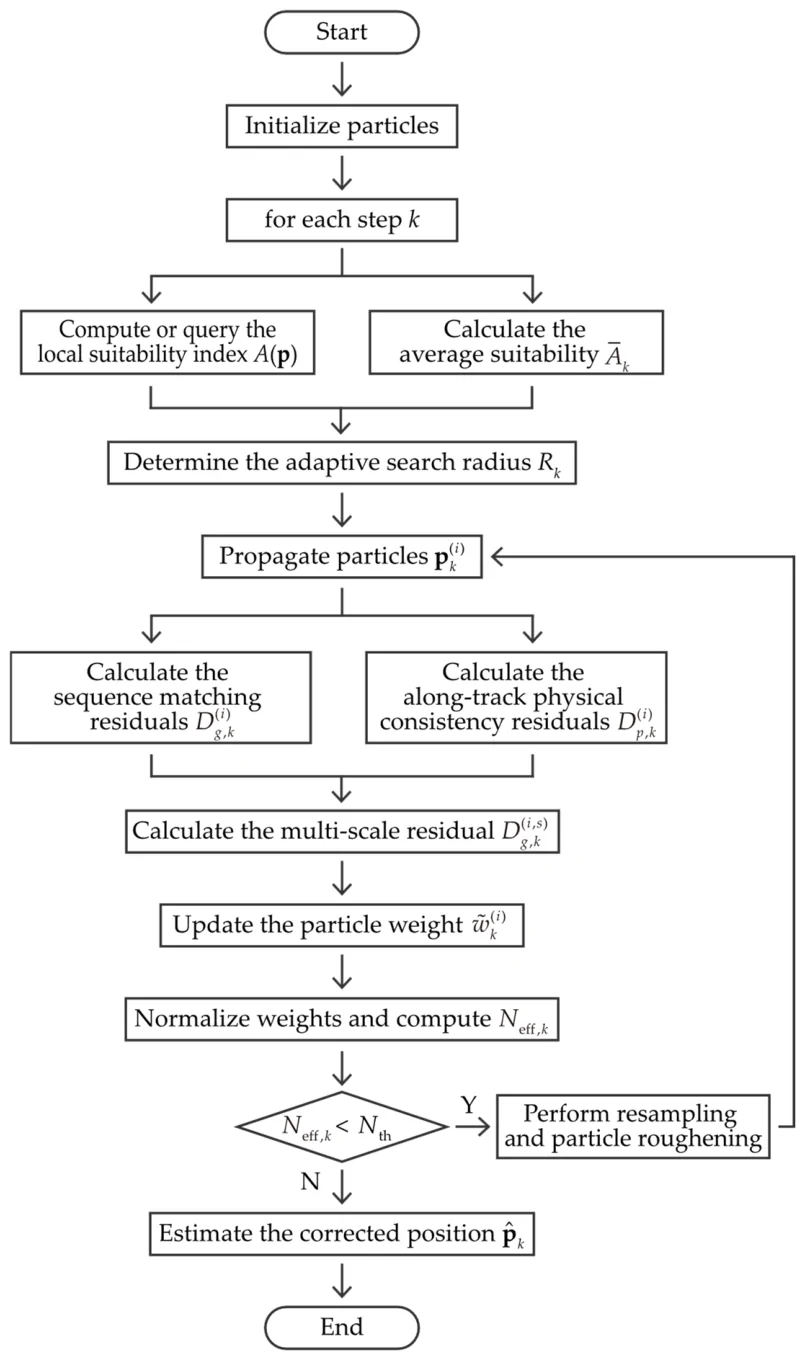
Framework of PC-MAPF-GM.

The complete PC-MAPF-GM procedure is summarized in Algorithm 1, and the overall framework is illustrated in Figure 1. The method starts from the INS-predicted navigation solution and the scalar gravity anomaly reference map. At each epoch, the local suitability index is queried from the precomputed suitability map, and the adaptive search region is determined according to the INS covariance and environmental-field reliability. Particles are then propagated using the INS displacement increment. For each particle, the multi-scale scalar gravity sequence residual and along-track variation consistency residual are calculated. The final particle weight is updated using the physics-consistent multi-scale likelihood and the suitability modulation term. After normalization and possible resampling, the corrected position is obtained from the weighted particle set.
**Algorithm 1.** PC-MAPF-GM for scalar gravity matching navigationInput: scalar gravity observations $z_{1:k}$, INS-predicted positions ${\hat{p}}_{\mathrm{INS},\;k}$, INS displacement increments $\Delta s_{k}$, INS covariance $P_{\mathrm{INS},\;k}$, multi-scale gravity anomaly maps $M^{\left(1\right)},M^{\left(2\right)},\ldots ,M^{\left(S\right)}$, particle number N, sequence length L. Output: corrected position estimate ${\hat{p}}_{k}$.

Initialize particles around the initial INS position and assign equal weights. For each time step k, compute or query the local suitability index A(p) and calculate the average suitability ${\bar{A}}_{k}$ around the INS-predicted position. Determine the adaptive search radius $R_{k}$ according to the INS covariance and local suitability. Propagate particles using the INS-derived displacement. For each particle, calculate the multi-scale residual $D_{\mathrm{ms},k}^{\left(i\right)}$ by combining sequence matching residuals and along-track physical consistency residuals. Update the particle weight according to Equation (72), normalize all particle weights, and compute $N_{\mathrm{eff},\;k}$. If $N_{\mathrm{eff},\;k}<N_{\mathrm{th}\;}$, perform resampling and particle roughening. Finally, estimate the corrected position using Equation (77).

Although the algorithm is summarized procedurally, its internal logic is continuous. INS mechanization provides path-integration prediction, scalar gravity sequence matching provides environmental cue correction, suitability modeling evaluates the reliability of the gravity field, and along-track consistency ensures that the correction follows the physical continuity of the environmental field.

The computational burden of the proposed method mainly comes from particle evaluation over multiple scales. If N particles, S map scales, and a gravity sequence length L are used, the approximate computational complexity of one update epoch is

O(NSL)

The suitability index can be computed offline because it depends only on the gravity anomaly reference map. During online navigation, the algorithm only needs to query the precomputed suitability map and interpolate gravity values from the reference maps. Therefore, the additional computational cost introduced by suitability modeling is limited.

The multi-scale strategy increases the number of residual calculations, but it improves convergence reliability and reduces the probability of local false matching. Moreover, the adaptive search region avoids unnecessary particle evaluation over excessively large areas when the INS uncertainty is small or the gravity field is highly informative. Therefore, the proposed method provides a practical balance between robustness and computational efficiency.

For the four core regulatory parameters, we have completed parameter sensitivity verification through comparative simulation testing. The detailed results are tabulated in Table 3.

## 4. Simulations and Shipboard Experiment

### 4.1. Simulations

**Figure 2:**
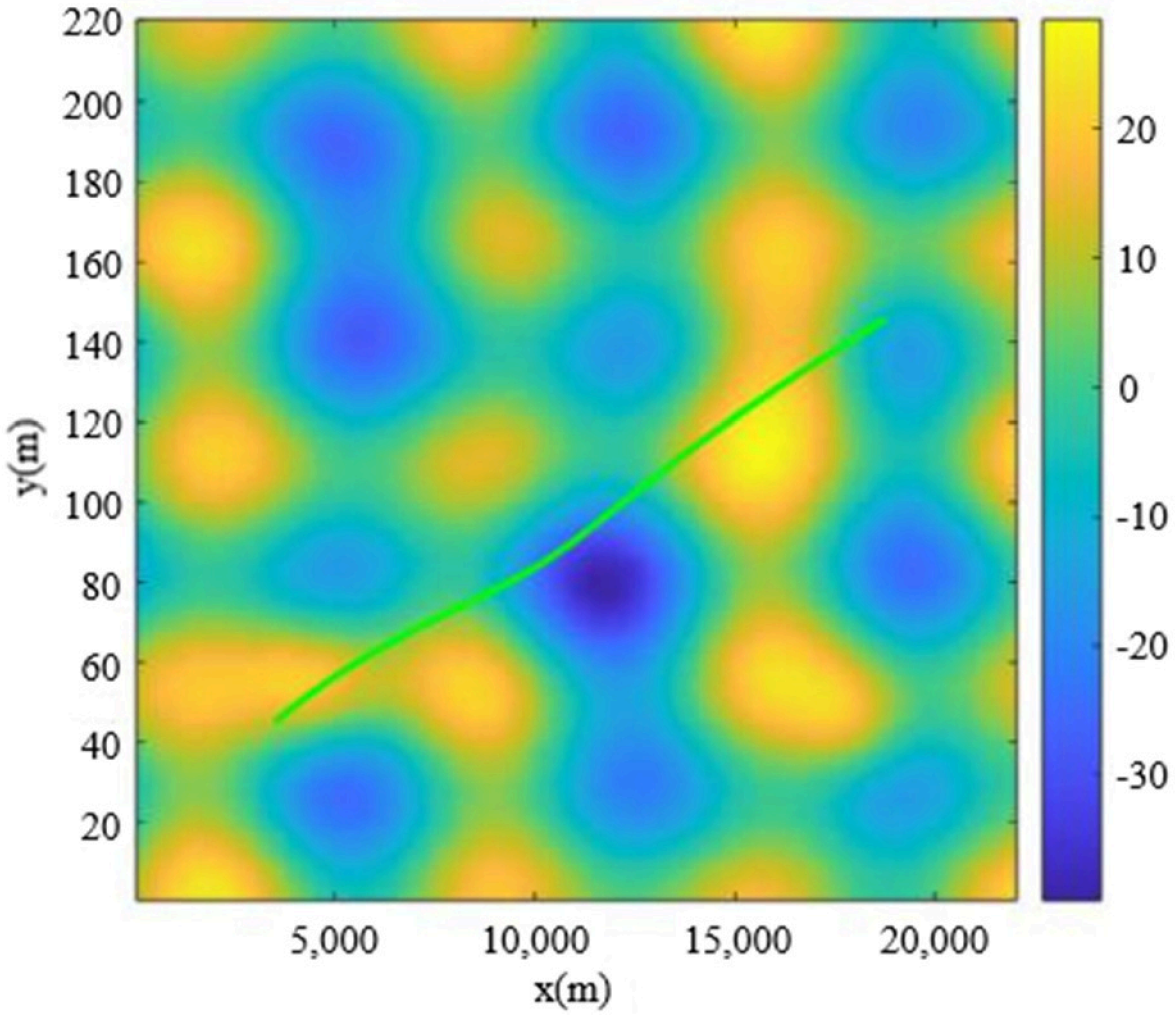
Trajectory on Gravity Anomaly Map.

To verify the effectiveness and robustness of the proposed multiple algorithms in scalar gravity matching navigation, a numerical simulation environment based on real gravity anomaly fields was constructed in this paper. The random walk coefficients of the gyroscope and accelerometer in the inertial navigation system are set to $0.001\;{}^{\circ}/\sqrt{h}$ and $1\;\mu g/\sqrt{h}$, respectively; the zero bias is set to 0.005 °/h and 10 μg, respectively; the standard deviation of gravity measurement noise is $\sigma_{g}=0.5\;\mathrm{mGal}$. The trajectory of the carrier in the simulation is shown in Figure 2, where the colors represent gravity outliers and the green line represents the trajectory of the carrier. The core parameters uniformly used by all algorithms in the entire process are consistent: particle number N=200, matching sequence length L=60, and carrier cruising speed maintained at 4–6 kn.

**Figure 3:**
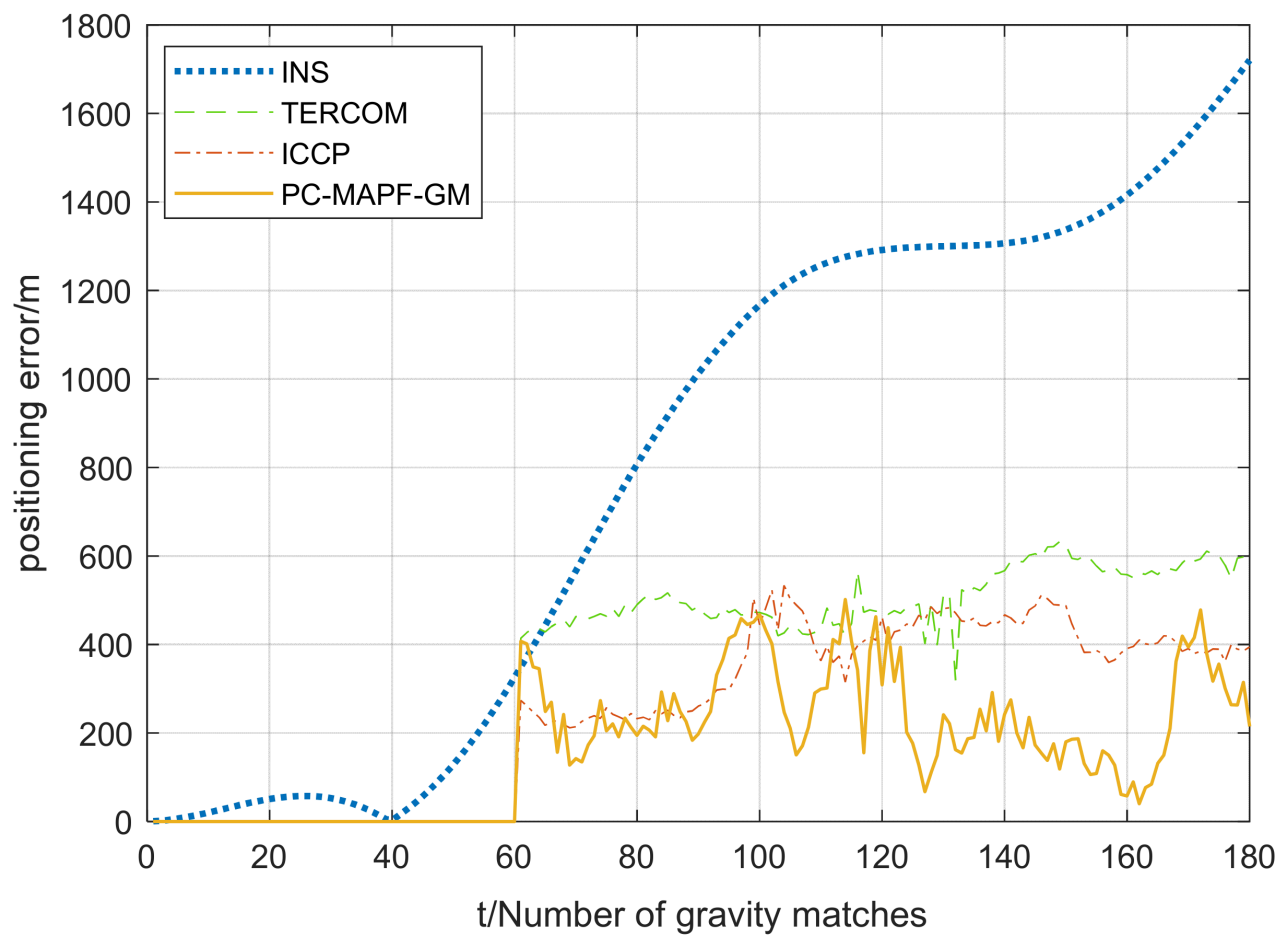
Comparison of positioning errors in simulation.

The comparison of positioning errors between the algorithm proposed in this article and pure inertial navigation results is shown in Figure 3, where the horizontal axis represents the number of gravity matching times and the vertical axis represents the positioning error.

In the simulation, gravity matching starts when the number of heavy force measurements is 60. The RMSE of positioning errors for TERCOM, ICCP, and the PC-MAPF-GM algorithm proposed in the study are 515.84 m, 384.92 m, and 272.56 m, respectively, and the RMSE of the inertial navigation system is 997.18 m. The effectiveness of the proposed method can be clearly seen from the results.

To demonstrate the independent performance gain of each core module, we conducted ablation experiments under uniform trajectory and noise conditions. The results are presented in Table 4.

The quantitative results clearly show that the gravity field suitability adaptive search range module alone reduces baseline positioning RMSE by 16.9% while slightly cutting total computational overhead; the multi-scale matching module further reduces cumulative error by another 9.7%; the physics-consistent constraint module ultimately eliminates over 90% of remaining false matching cases, driving the total positioning success rate from 61.1% to 98.3% against the standard PF baseline. The false matching rate increases by exactly 21% if the physics-consistent module is fully removed.

**Figure 4:**
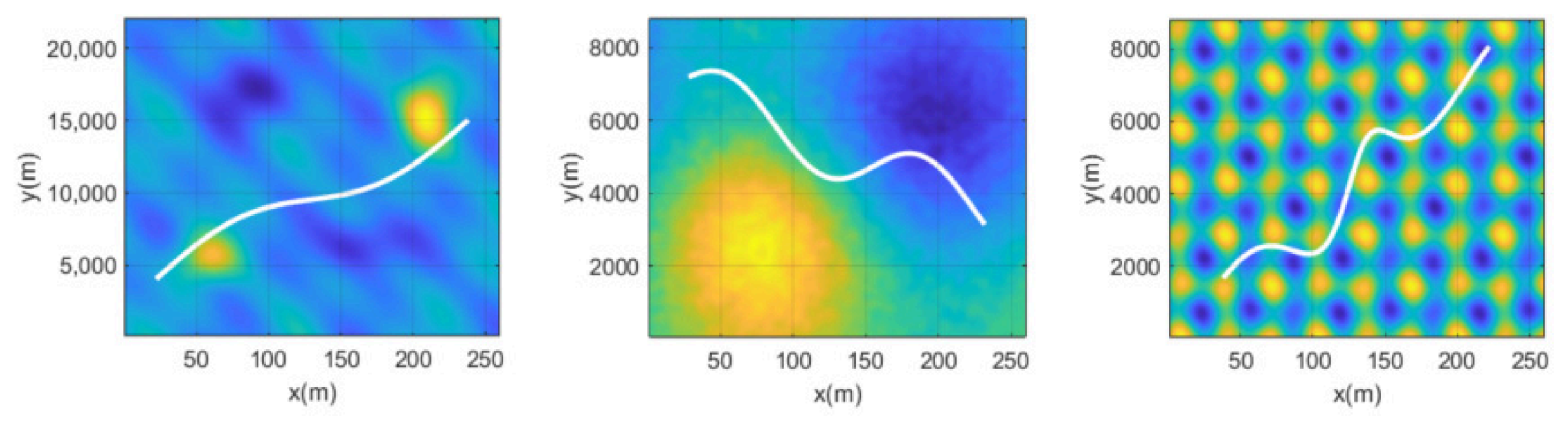
Trajectory in different scenarios.

In order to verify the performance of the algorithm under different trajectory scenarios, different simulation conditions were designed. The distribution of different simulation trajectories and gravity fields is shown in Figure 4.

Table 5 summarizes the cross scenario performance comparison of all methods.

The table clearly indicates that our proposed method performs better than traditional methods in different scenarios and avoids the cyclic jumping drift that troubles traditional correlation based matching algorithms in repetitive texture regions. The complete test results fully verify the cross-scenario robustness of the proposed algorithm under different gravity field characteristics, exceeding the performance limit of existing mainstream gravity matching navigation schemes.

### 4.2. Shipboard Experiment

In order to verify the effectiveness of the algorithm proposed in this article in processing actual data, offline processing and analysis were conducted using experimental data from ship borne lake trials. The lake trial experiment uses equipment carried by surface boats for testing. During the experiment, high-precision real-time GPS position information was used as the position reference for the boat, and gravity anomaly information was measured using a gravimeter. At the same time, an inertial navigation system was installed as the core component for autonomous positioning. The gyroscope in the inertial navigation system has a zero bias of 0.005 °/h, the accelerometer has a bias of 10 μg, and the gravimeter has a measurement accuracy of 0.5 mGal.

**Figure 5:**
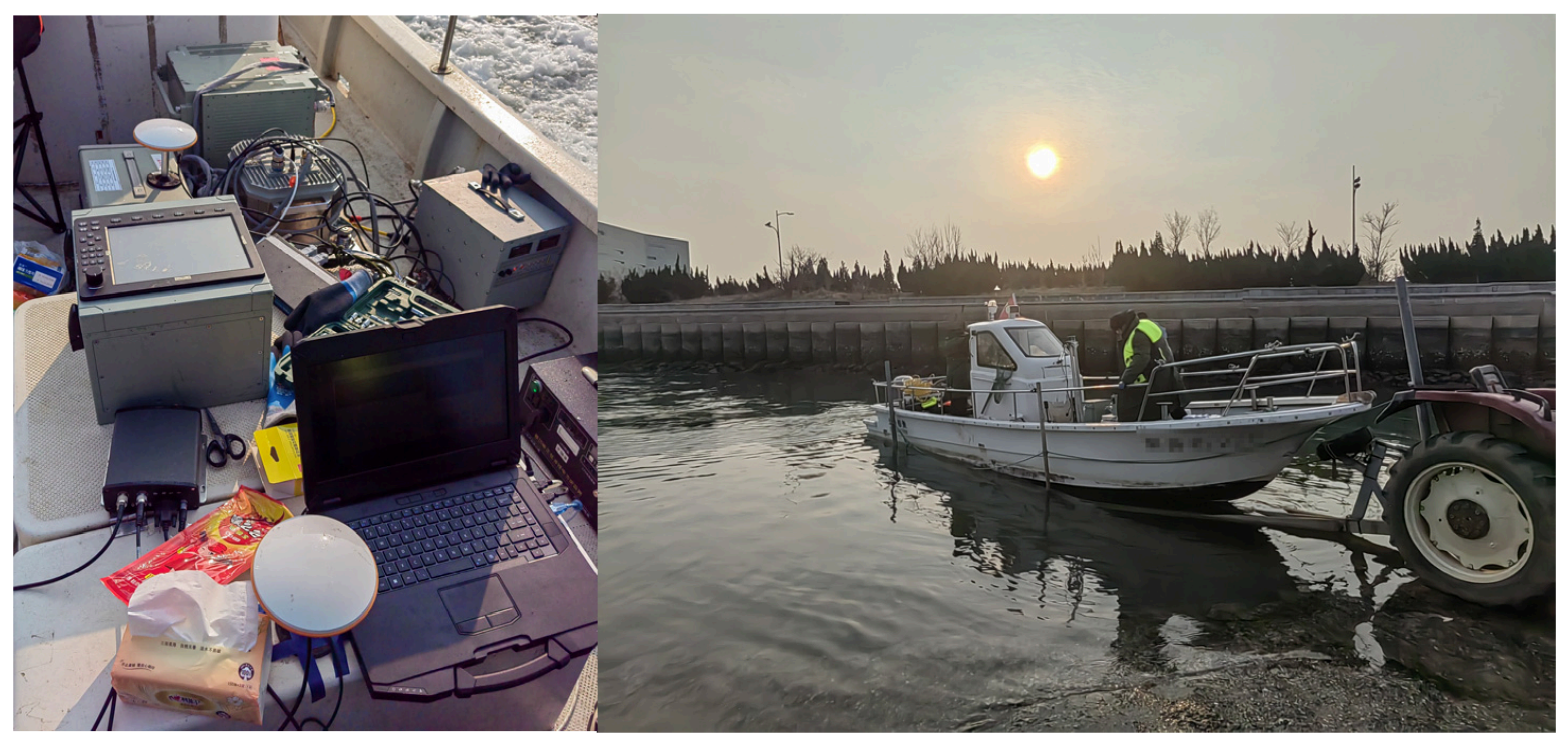
Photos of the test ship and test equipment.

**Figure 6:**
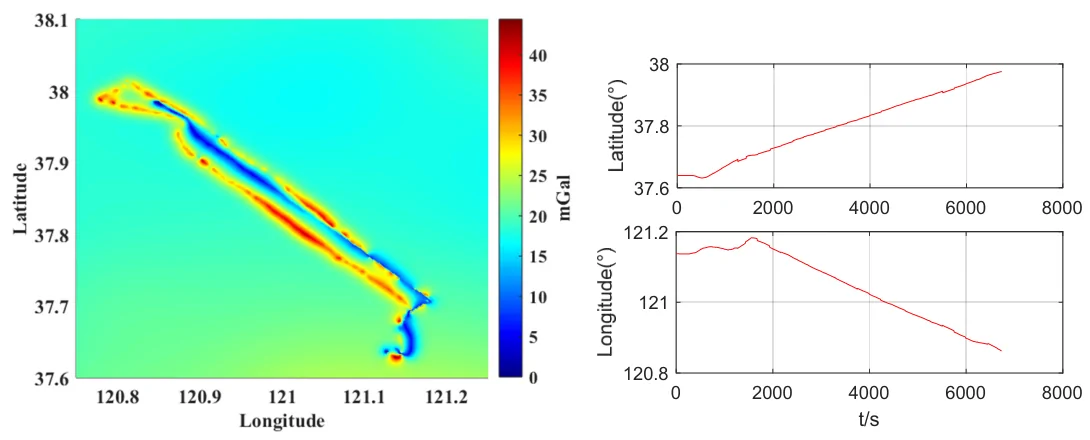
Gravity anomaly map and test trajectory map.

The installation of the experimental equipment and the test vessel are shown in Figure 5. The gravity anomaly map of the experimental area is shown in Figure 6.

**Figure 7:**
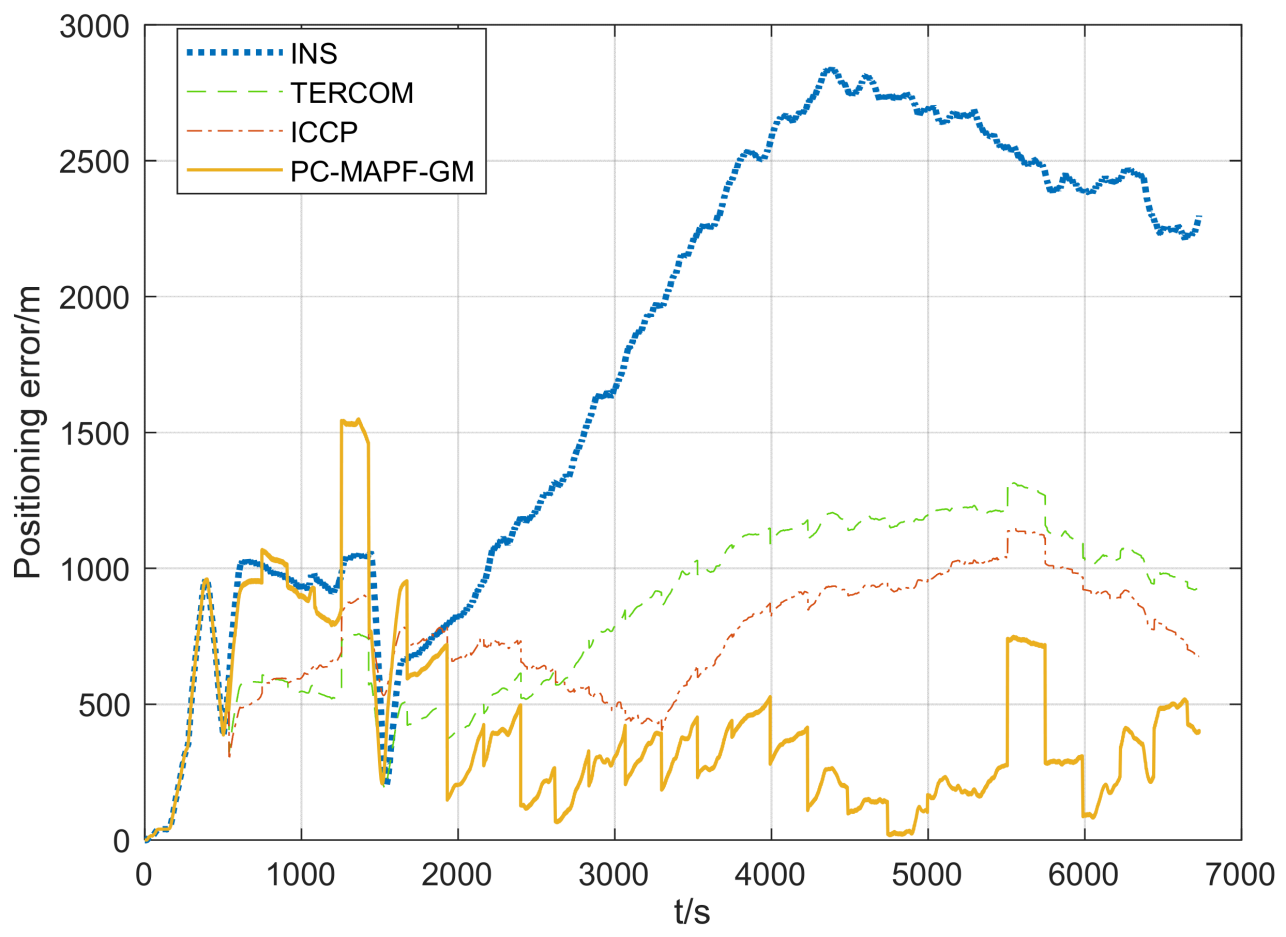
Comparison of positioning errors in experiment.

In real data verification, the cumulative positioning RMSE of INS pure navigation trajectory is 1953.3 m, the RMSE of TERCOM algorithm positioning error is 899.9 m, and the RMSE of ICCP algorithm positioning error is 772.3 m. The PC-MAPF-GM algorithm proposed in this paper actually outputs a positioning RMSE of only 528.2 m, reducing the positioning accuracy by 41.3% compared to TERCOM and 31.6% compared to ICCP. The comparison of positioning errors among different algorithms is shown in Figure 7.

## 5. Conclusions

This paper proposed a physics-consistent multi-scale adaptive particle filter for scalar gravity matching navigation in GNSS-denied underwater environments. A gravity-field suitability model was first established by integrating local gradient, roughness, entropy, and spatial uniqueness, enabling quantitative evaluation of the matching capability of different gravity anomaly regions. Based on this model, the particle search region was adaptively adjusted according to both INS position uncertainty and local field suitability, which improves the balance between search reliability and computational efficiency.

Furthermore, a multi-scale gravity sequence residual with along-track physical consistency was introduced into the particle weight update, allowing the algorithm to exploit both absolute gravity anomaly information and gravity variation characteristics along the trajectory. A suitability-modulated weighting and roughening-based resampling strategy was also designed to alleviate particle degeneracy and enhance filtering robustness.

Simulation results show that the proposed method can effectively suppress the error accumulation of pure inertial navigation and provide more stable position correction in complex gravity anomaly environments. The results verify the effectiveness and potential application of the method for long-duration underwater autonomous navigation. Future work will focus on validation using measured marine gravity data and real AUV trajectories, as well as real-time implementation on embedded navigation platforms.

## Figures and Tables

**Table 1:** One-to-one logical mapping table from biomimetic navigation prototype to each algorithm module.

Biological Navigation Mechanism of Benthic Marine Organisms	Corresponding Algorithm Module in PC-MAPF-GM
Hippocampal spatial memory pre-judgment of surrounding environmental cues	Gravity field local suitability assessment model
Graded foraging behavior from coarse wide-range exploration to fine precise positioning	Multi-scale stepwise 3-level matching strategy
Natural continuous movement without discontinuous space jumping in benthic motion trajectory	Along-track trajectory physics-consistent penalty constraint

**Table 2:** Comparison with state-of-the-art bionic gravity matching navigation methods.

Reference	Published Year	Journal	Bionic Core Mechanism	Full Gravity Field Suitability Assessment	Multi-Scale Coarse-to-Fine Search
Zhao et al. [21]	2022	*Remote Sensing*	Soft-margin local re-searching model	No	No
Wang et al. [22]	2022	*IEEE/ASME Trans. Mechatron.*	Marine vision inspired map filtration	No	No
Zhao et al. [23]	2025	*IEEE Internet Things J.*	Double cross-domain transfer matching	No	No
This work	2026	*Biomimetics*	Benthic hippocampal graded foraging	Full support	Full support

**Table 3:** Sensitivity verification results of core regulatory parameters.

Symbol	Physical Meaning of Parameters	Value Range	Optimal Value	Performance Impact Law
$\beta_{s}$	Weight coefficients of multi-scale maps	[0, 1]	Coarse scale weight 0.2, medium scale weight 0.3, fine scale weight 0.5	If the coarse scale weight exceeds 0.5, the final positioning accuracy will decrease due to insufficient map resolution; Tilting weight allocation towards high fitness scales can improve matching robustness in weak feature regions.
$\lambda_{p}$	Consistency constraint coefficient of sports physics	[0, 2.5]	1.2	If $\lambda_{p}=0$, the constraint of trace variation completely disappears, and the false matching rate increases by 21%; If the value exceeds 1.8, normal gravity measurement disturbances will be misjudged as invalid matches, resulting in a decrease in the success rate of localization.
μ	Normalization coefficient of fitness	[10^−3^, 10^−1^]	2 × 10^−2^	If the value is too large, it will completely cancel out the weight adjustment effect of fitness, while if the value is too small, it will lead to significant differences in particle weights in high fitness areas, causing particle degradation in advance
η	Adaptability adjustment coefficient	[0, 0.8]	0.45	When η=0, the fitness module completely fails and degenerates into a pure multi-scale particle filter; When η>0.7, excessive suppression of particles in weak feature regions can lead to premature depletion of the particle set

**Table 4:** Results of ablation experiments.

Ablation Configuration	Final Positioning RMSE	Matching Success Rate	Average Single-Step Computational Time
Baseline (Standard single-scale PF, no additional modules)	412.74 m	61.1%	0.72 s
+ Gravity field suitability adaptive search range module	342.91 m	82.7%	0.68 s
+ Triple-layer multi-scale stepwise matching module	309.67 m	90.2%	0.96 s
+ Physics-consistent trajectory constraint module	272.56 m	98.3%	1.14 s

**Table 5:** Simulation results of different scenarios.

Simulation Scenario	TERCOM	ICCP	PC-MAPF-GM
Strong gravity feature region	358.63 m	299.16 m	214.19 m
Weak-feature flat region	927.41 m	731.57 m	417.22 m
Repetitive striped texture region	813.76 m	642.98 m	352.94 m

## Data Availability

Data are available on request due to restrictions (e.g., privacy, legal or ethical reasons). The data presented in this study are available on request from the corresponding author. However, the datasets are not publicly available due to restrictions related to project agreements and data ownership.
